# Transcriptome Analysis of Host Inflammatory Responses to the Ectoparasitic Mite *Sarcoptes scabiei* var. *hominis*

**DOI:** 10.3389/fimmu.2021.778840

**Published:** 2021-12-01

**Authors:** Huma Shehwana, Sadaf Ijaz, Abeera Fatima, Shelley Walton, Zafar Iqbal Sheikh, Waseem Haider, Shumaila Naz

**Affiliations:** ^1^ Department of Biological Sciences, National University of Medical Sciences, Rawalpindi, Pakistan; ^2^ Research Centre for Modelling & Simulation, National University of Science and Technology, Islamabad, Pakistan; ^3^ Inflammation and Healing Research Cluster, School of Health and Sport Sciences, University of the Sunshine Coast, Maroochydore, QLD, Australia; ^4^ Department of Dermatology, Pak-Emirates Military Hospital, Rawalpindi, Pakistan; ^5^ Department of Biosciences, COMSATS University Islamabad, Islamabad, Pakistan

**Keywords:** RNA-seq data analysis, *Sarcoptes scabiei.* var. *hominis*, inflammatory responses, scabies, differentially expressed genes, JAK-STAT pathways

## Abstract

Scabies, a human skin infestation caused by the ectoparasitic mite *Sarcoptes scabiei* var. *hominis*, affects more than 200 million people globally. The prevailing knowledge of the disease process and host immune response mechanisms is limited. A better understanding of the host-parasite relationship is essential for the identification of novel vaccine and drug targets. Here we aimed to interrogate the transcriptomic profiles of mite-infested human skin biopsies with clinical manifestations of ordinary scabies subjects (“OS”; n = 05) and subjects naive to scabies (“control”; n = 03) using RNASeq data analysis. A combined clustering, network, and pathway mapping approach enabled us to identify key signaling events in the host immune and pro-inflammatory responses to *S. scabiei* infestation. The clustering patterns showed various differentially expressed genes including inflammatory responses and innate immunity genes (DEFB4A, IL-19, CXCL8, CSF3, SERPINB4, S100A7A, HRNR) and notably upregulation of the JAK-STAT pathway in scabies-infested samples. Mite-infested human skin biopsies (GSE178563) were compared with an *ex-vivo* porcine infested model (E-MTAB-6433) and human skin equivalents (GSE48459). Marked enrichment of immune response pathways (JAK-STAT signaling, IL-4 and IL-13 pathway, and Toll receptor cascade), chemokine ligands and receptors (CCL17, CCL18, CCL3L1, CCL3L3, CCR7), and cytokines (IL-13 and IL-20) were observed. Additionally, genes known for their role in psoriasis and atopic dermatitis were upregulated, e.g., IL-19. The detailed transcriptomic profile has provided an insight into molecular functions, biological processes, and immunological responses and increased our understanding about transcriptomic regulation of scabies in human.

## Introduction

Scabies is a common contagious human skin infestation caused by ectoparasite *Sarcoptes scabiei* var. *hominis* (*S. scabiei* var. *hominis*) (1, 2). World Health Organization (WHO) listed scabies as one of the top 50 epidemics worldwide (3, 4) with an infection rate as high as 50–80% in certain populations, and up to 10% of the global human population infected (5). Infestation by the mite almost always leads to the development of localized skin inflammation, itching, and burrow formation; rash and itch possibly being an indicator of host immune response with features of both type I and type IV hypersensitivity reactions (6).

The initial immune response towards the mite and its products, by different hosts, consists of keratinocytes, neutrophils, Langerhans cells, and macrophages (7, 8), which initiate an inflammatory and immune reaction (9). Granulocytes are engaged in boosting the immunity of the host against a variety of parasites by initiating immunomodulation and producing cytokines and chemokines, which direct the immune response. Upon recruitment to infection site/draining lymph nodes, eosinophils, mast cells, and basophils produce IL-4 and /or IL-13 (10). In a study reported by Walton et al. in 2008, skin biopsies taken from scabies patients having crusted lesions showed large numbers of lymphocytes, monocytes, macrophages, and eosinophils in the dermis, with increased levels of IgE in the blood samples. Similar findings have also been reported in the skin, upper airways, and lung cellular infiltrates present in chronic allergic inflammation (11).

The immune response to *S. scabiei* consistently shows increased levels of CD4+ or CD8+ T cells. CD4+ T cells mostly dominate the infiltrates of skin biopsy lesions in ordinary scabies, with a 4:1 ratio of CD4+ to CD8+ T cells (12, 13). This is similar to studies of inflammatory cells in the biopsies of skin lesions from patients with atopic dermatitis, which demonstrate significantly greater number of infiltrating CD4+ lymphocytes compared with CD8+ subtypes (14). In comparison, the skin inflammatory response in patients with crusted scabies predominantly comprises CD8+T cells (6, 12).

Scabies mites are highly host-specific and commonly produce a temporary, self-limiting reaction in the non-preferred host (15). Previous animal studies of host immune response in scabies have used *S. scabiei* var. *canis* whole mite antigen extracts in rabbits and mice, but interpretation of these studies is mystified probably by a mismatched host-parasite system (16). However, various studies have consistently shown that the immune response of the host to ordinary scabies is a Th1 cell-mediated protective type and to the crusted variety of the scabies is a non-protective Th2 allergic response (6, 17, 18). In addition, Crusted Scabies (CS) patients have shown increased production of Th2 cytokines IL-4, IL-5, and IL-13 and decreased secretion of the Th1 cytokine IFN-γ as compared to OS (19).

The prevailing knowledge of the disease processes and host immune response mechanisms is limited, and a better understanding of the host-parasite relationship is essential to develop a novel vaccine or a drug (20). mRNA profiling will provide new insights into the events/signaling mechanisms leading to the development of immune and pro-inflammatory responses in scabies. Recent gene expression findings using microarray in sheep scab, psoriasis, and atopic dermatitis indicate a direct relationship between gene expression and disease phenotype (19, 21).

The pattern of gene expressions is incredibly useful in understanding the various pathways involved in the immune response to scabies. Scabies, being an inflammatory allergic disease, shares similar clinical symptoms and immune and inflammatory responses to psoriasis, atopic dermatitis, and sheep scab (22). Further understanding of the pathways involved in immune responses to scabies might also be useful in understanding the immunogenic responses to other inflammatory allergic diseases. In this study, we have examined the transcriptomic profiles of skin RNA samples of scabies patients and healthy controls and identified the key signaling events in the host immune and pro-inflammatory responses to *S. scabiei* infestation. As we did not find any transcriptome profiling using human skin biopsies, we have performed comparative analysis on artificially infested porcine samples, i.e., E-MTAB-6433 (23) and human skin equivalents, i.e., GSE48459 (24), with our model to identify a robust gene signature.

## Materials and Methods

**Figure 1:**
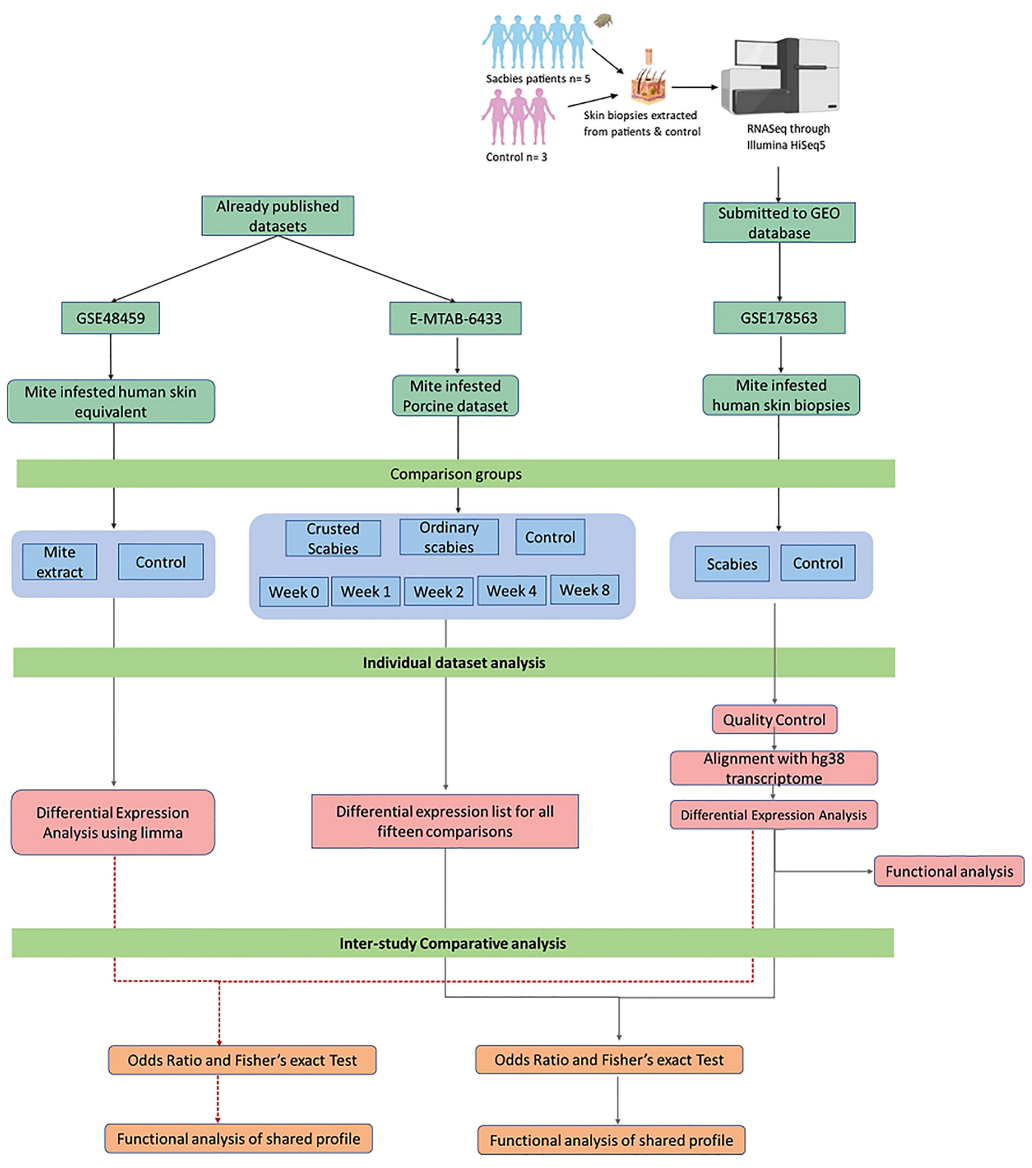
Workflow of the project.

The workflow of the methodology to examine the transcriptomic profiles of skin RNA samples of scabies patients and healthy controls and to identify the key signaling events in the host immune to *S. scabiei* var. *hominis* infestation is given in **Figure 1**.

### Ethical Approval

Ethical approval for collection of human skin biopsies was granted by the Chairman, Institutional Review Board, and Ethics Committee of the National University of Medical Sciences (NUMS), Rawalpindi, Pakistan, after compliance to the observations of the two reviewers, under letter No. NUMS/P(VC) – 17/R&D/ORIC/IRB&EC approved on November 9, 2017.

### Sample Collection

Skin biopsies were collected from patients clinically diagnosed to have been suffering from scabies by visiting the Department of Dermatology in Pak-Emirates Military Hospital (PEMH), Rawalpindi, Pakistan, between January to March 2018. The skin biopsies were collected after obtaining written informed consent from a total of eight participants in two categories: ordinary scabies subjects (“OS”; n = 05) and individuals naive to scabies and allergy with no known exposure to scabies or allergy (control”; n = 03). Before biopsy, the skin was thoroughly cleaned and a small injection of a local anesthetic (Lignocaine Injection) to numb the skin was made. The 3 mm punch skin biopsies were collected from lesional sites of each patient. The cases of OS were confirmed by clinical observation and positive identification of mites and mite parts, collected from skin scrapings, under the microscope (1 mite or mite part/scraping). The data about age, gender, locality, and duration of infestation was obtained by questionnaire and recorded. Skin biopsies were stored in RNAlater (Thermo Fisher Scientific, USA) at −20°C till shipping on ambient temperatures to Novogene, China, for Next-Generation Sequencing (NGS).

### RNA Extraction, Library Preparation, and RNA Sequencing

mRNA extraction of skin biopsies was performed by Novogene, Beijing (China). The preliminary Quality Control (QC) check of mRNA samples was done on agarose gel electrophoresis. The quantity and purity of the RNA samples were checked using a Qubit fluorometer, while Agilent 2100 was used to check the RNA integrity (RIN) of the samples. Library preparation and mRNA sequencing were performed as per the standard procedure. Sequencing was done on an Illumina HiSeq4000 instrument, generating 150 base-pair (bp) PE sequencing data.

### RNA-Seq Data Analysis

Quality control of raw RNA-seq data was evaluated using FASTQC (http://www.bioinformatics.babraham.ac.uk/projects/fastqc/). Paired-end reads were aligned to the human reference transcriptome (hg38) obtained from UCSC genome browser using Burrows-Wheeler Alignment (BWA-mem algorithm) (25). Transcript-specific count data of aligned samples were obtained using the HTSeq-count tool (26). Differential expression analysis between ordinary scabies and control samples was performed on raw count data by using the exact test from the edgeR 3.34 package (27).

### Pathway Enrichment Analysis and Gene Set Enrichment Analysis

Genes exhibiting significant differences between both groups (p<0.05 and logFC >absolute 2) were used for pathway enrichment analysis using Database for Annotation, Visualization, and Integrated Discovery (DAVID) online tool version 6.8 (28, 29). Upregulated and downregulated gene lists were separately queried using Gene Ontology (Biological Processes, Molecular Function) and Kyoto Encyclopedia of Genes and Genomes (KEGG) databases in DAVID online tool. Terms with FDR (BH) <0.05 were considered as significantly enriched functional classification and are represented in the form of plots.

In addition, gene set enrichment analysis was performed using GSEA software v. 4.1.0 (Broad Institute, MIT, Cambridge, MA, USA) (30). Raw gene count data were log-transformed, and genes with no or little variation (SD<0.25) were removed prior to GSEA analysis. GSEA software was run using MSIGDB Gene Ontology–Biological processes gene set database with the customized settings (1,000 iterations, gene set permutation, and 149 seed value). Significant terms in the enrichment map (FDR<0.1 and p<0.005) were visualized using cystoscope software.

### Comparative Analysis With E-MTAB-6433

For comparative analysis with E-MTAB-6433, time-series based differential gene expression lists for OS *vs.* C, CS *vs.* C, and CS *vs.* OS were retrieved from database (23). We obtained Porcine-Human orthologs gene lists from the ensemble Biomart database by using the R package biomaRt 3.13 (31). Differentially expressed gene lists from the porcine studies were merged with our human-infested skin biopsy dataset using ortholog lists (edgeR results; p<0.05). Odds ratio and fishers exact test were used to find the association of human skin biopsy scabies dataset with each combination of time-series Pig dataset. Correlation analysis was performed using the stat_cor function (ggpubr package) in R.

### Comparative Analysis With GSE48459

We downloaded RNA normalized data of human skin equivalent gene expression data (GSE48459) from the GEO database (24). GSE48459 is based on Affymetrix Human Gene 1.0 ST Array. Probe to gene annotation was retrieved from Ensembl Biomart. Differential expression analysis was performed on live mites *vs.* control using the Limma package (32). To represent each gene with one probe, duplicate probes were removed by selecting a single probe with the smallest p-value. GSE48459 was merged with our human skin biopsies dataset using Ensembl gene IDs. Significantly differentially expressed profiles (p-value<0.05) from both datasets, i.e., (GSE48459—live mites *vs.* untreated human skin equivalents), and human skin biopsies (scabies *vs.* no-lesion) were compared using odds ratio and fisher’s exact test. Correlation analysis was performed using the statcor function (ggpubr package).

### Graphs Visualization

Venn diagrams were generated using vennerable package. Graphs were plotted using ggplot2 package. All analysis was performed in R.

## Results

In the present study, we have collected skin biopsies of five patients including four males and one female. The skin biopsy was taken from confirmed scabies mite-infested patients with clinical signs and symptoms of 1–2 weeks. In addition, the skin biopsies of three naïve individuals with no history of skin allergy were also included in the analysis as controls to avoid any confounding in inflammatory host immune responses and allow data comparison and interpretation with the porcine model. The patient and control samples data along with some demographic details is provided in **Supplementary Table 1**. RNA extracted from all eight skin biopsy samples showed a good RIN value ranging between 7.6 to 8.6 (**Supplementary Figure 1**).

### Identification of Dysregulated Gene Expression Profile in Infested Samples

**Figure 2:**
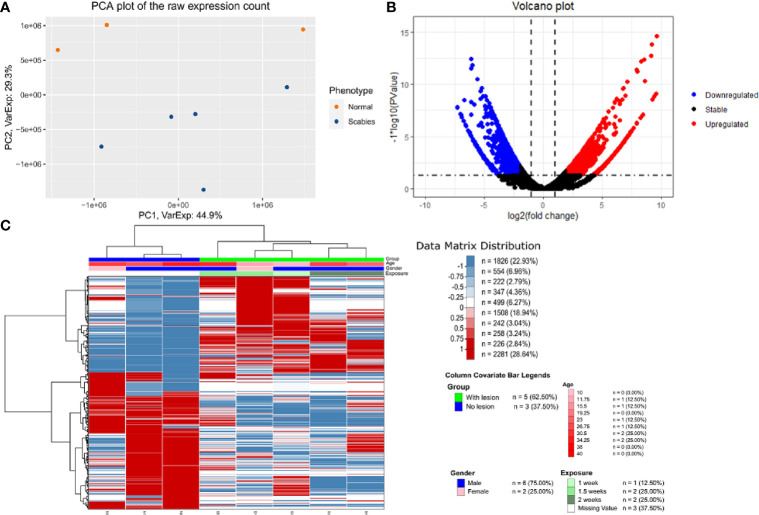
Transcriptome profile of human skin biopsies infested with mite (*S. scabiei* var. *hominis*) **(A)** PCA plot using raw RNA-seq expression counts of eight samples (three control and five OS samples). **(B)** Volcano plot of differential expression profile of all genes with upregulated (p < 0.05 and logFC>2) and downregulated (p < 0.05 and logFC < 2) genes shown in red and blue respectively. **(C)** NGCHM plot of top 1,000 differentially expressed genes using unsupervised hierarchical clustering in both directions (rows and columns). Covariates include age, gender, and duration of exposure and diseased group (With lesion represents OS samples, and No lesion represents control samples).

We carried out paired-end RNA-sequencing for freshly frozen skin biopsy samples. RNA-seq data exhibited good quality with almost 30 million reads per sample. Approximately 90–96% raw reads successfully mapped to human reference transcriptome hg38 (**Supplementary Table 2**). An early exploratory PCA plot using all raw counts in the dataset showed an overall distinct transcriptomic pattern of raw counts from scabies and control sample profiles (**Figure 2A**). Differential expression analysis revealed that a large number of genes were significantly dysregulated, with 1,618 upregulated and 1,636 downregulated genes (p-value<0.05 and absolute logFC>2) in mite-infested samples compared to the control group (**Figure 2B**). The top thousand differentially expressed genes were visualized using unsupervised clustering. Clustering patterns showed that upregulated and downregulated genes grouped together in two distinct clusters. Notably, sample-wise clustering also revealed homogenous gene expression within each subgroup (three control and five mite-infested samples) with the greatest variability across the group. Within the mite-infested group, samples showed partial clustering with respect to the duration of exposure, hence indicating that transcriptomic changes mediated by mite infestation might be a time-dependent mechanism (**Figure 2C**). Top upregulated genes displayed a role in inflammatory responses and innate immunity (DEFB4A, IL-19, CXCL8, CSF3, SERPINB4, S100A7A, HRNR), keratinization (KRT9, KRT6C, EGR4, SPRR3, LCE3A, SPRR2A, LCE3A), and a gene involved in desquamation processes in skin (SPINK9); antigen recognition genes, binding of immunoglobulin genes (IGLV6-57, IGHV3-30, IGLV4-69, IGHEP1), and T-cell receptor gene (TRBV13) were the top downregulated in infested samples. Furthermore, genes involved in detoxification and homeostasis (FUT9 and UGT2B28) and hydrolase and peptidase activity (PM20D1) also showed more than five-fold downregulation in diseased samples. Interestingly, AADACL3 was remarkably downregulated. The chromosomal locus of the AADACL3 gene has been previously reported in recurrent Norwegian scabies samples (33). The top 20 up- and downregulated genes are listed in **Table 1**.

**Table 1:** Top 20 upregulated and downregulated genes in mite-infested human skin biopsies (n=5) with respect to control samples (n = 3).

	Ensembl Gene ID	HGNC Symbol	logFC	logCPM	PValue	FDR
**Upregulated genes**	ENSG00000171711	DEFB4A	12.76	3.96	1.71E-15	2.34E-11
	ENSG00000142224	IL19	10.76	6.10	4.04E-16	8.35E-12
	ENSG00000206073	SERPINB4	10.58	6.85	1.93E-16	7.98E-12
	ENSG00000171403	KRT9	9.62	11.71	2.27E-15	2.34E-11
	ENSG00000135625	EGR4	9.58	0.81	8.08E-10	1.59E-06
	ENSG00000166670	MMP10	9.45	0.68	1.37E-09	2.37E-06
	ENSG00000108342	CSF3	9.26	0.50	2.93E-09	4.02E-06
	ENSG00000170465	KRT6C	9.19	9.90	1.42E-14	1.17E-10
	ENSG00000184330	S100A7A	9.16	4.89	1.75E-13	1.20E-09
	ENSG00000204909	SPINK9	8.63	1.51	4.55E-11	1.25E-07
	ENSG00000169429	CXCL8	8.59	4.67	4.33E-13	2.23E-09
	ENSG00000171450	CDK5R2	8.44	−0.28	7.48E-08	5.33E-05
	ENSG00000163209	SPRR3	8.40	−0.31	8.86E-08	5.90E-05
	ENSG00000197915	HRNR	8.32	6.41	6.73E-13	3.09E-09
	ENSG00000258397	BCAR1P1	8.26	−0.46	1.52E-07	8.71E-05
	ENSG00000185962	LCE3A	7.97	5.19	5.77E-12	1.83E-08
	ENSG00000215151	ABCD1P2	7.96	−0.74	4.88E-07	2.26E-04
	ENSG00000241794	SPRR2A	7.94	6.49	3.72E-12	1.28E-08
	ENSG00000259098		7.92	−0.77	5.69E-07	2.55E-04
	ENSG00000226807	MROH5	7.65	−1.01	1.57E-06	5.51E-04
**Downregulated genes**	ENSG00000211640	IGLV6-57	−7.29	−1.86	1.57E-08	1.47E-05
	ENSG00000270550	IGHV3-30	−7.25	−1.89	1.83E-08	1.64E-05
	ENSG00000211637	IGLV4-69	−7.02	−2.07	6.86E-08	5.06E-05
	ENSG00000273018	FAM106A	−6.98	−2.10	8.20E-08	5.64E-05
	ENSG00000240708	LINC02030	−6.91	−2.15	1.43E-07	8.55E-05
	ENSG00000251027	LINC01950	−6.76	−2.27	3.16E-07	1.61E-04
	ENSG00000253692	IGHEP1	−6.69	−1.14	3.02E-09	4.02E-06
	ENSG00000135226	UGT2B28	−6.61	−2.36	7.48E-07	3.12E-04
	ENSG00000229453	SPINK8	−6.54	−2.42	9.39E-07	3.66E-04
	ENSG00000235584		−6.39	−2.52	2.47E-06	7.96E-04
	ENSG00000187481	HSD3BP1	−6.38	−2.53	2.47E-06	7.96E-04
	ENSG00000205456	TP53TG3D	−6.29	−2.59	4.16E-06	1.18E-03
	ENSG00000223342		−6.24	−2.64	4.16E-06	1.18E-03
	ENSG00000276405	TRBV13	−6.15	−2.69	7.21E-06	1.86E-03
	ENSG00000172461	FUT9	−6.12	−1.55	8.07E-08	5.64E-05
	ENSG00000124935	SCGB1D2	−6.10	6.65	3.68E-13	2.17E-09
	ENSG00000277010		−6.10	−1.01	5.14E-09	6.06E-06
	ENSG00000188984	AADACL3	−6.09	1.77	2.90E-12	1.09E-08
	ENSG00000152591	DSPP	−6.08	−2.71	9.61E-06	2.28E-03
	ENSG00000162877	PM20D1	−6.02	3.05	1.44E-12	5.92E-09

### Functional Impact of Dysregulated Gene in Infested *vs.* Control Samples

**Figure 3:**
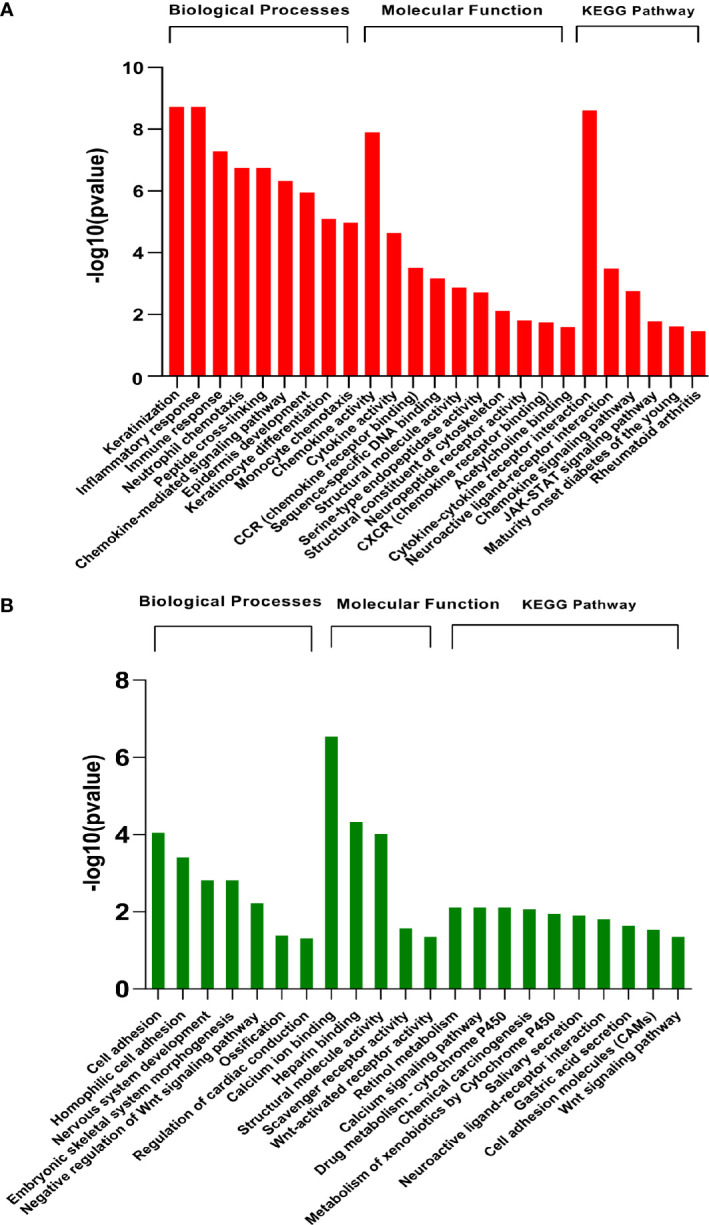
Pathway enrichment analysis of differentially expressed genes (GSE178563). Significantly **(A)** upregulated and **(B)** downregulated pathways (FDR<0.05) enriched in differentially expressed genes using Gene Ontology biological processes, molecular function, and KEGG database by DAVID web tool.

Functional analysis was conducted at two levels, i.e., pathway enrichment analysis using differentially expressed genes (p<0.05 and abs logFC>2) and Gene Set Enrichment Analysis (GSEA) using raw counts data. The enriched pathways in differentially expressed genes are shown in **Figure 3**. As expected, the results showed that pathways associated with skin development, including keratinization, epidermis development, and keratinocyte differentiation, were highly upregulated in the diseased samples. In addition, early immune-related responses including cytokine activity, chemokine signaling pathways, monocyte chemotaxis, and chemokine receptor binding are also upregulated in infested transcriptomic profiles. Notably, JAK-STAT pathway is upregulated in scabies-infested samples. Cell adhesion, negative regulation of Wnt signaling, calcium ion binding, scavenger receptor activity, retinol metabolism, and developmental processes were downregulated on the onset of infestation (**Figure 3**).

**Figure 4:**
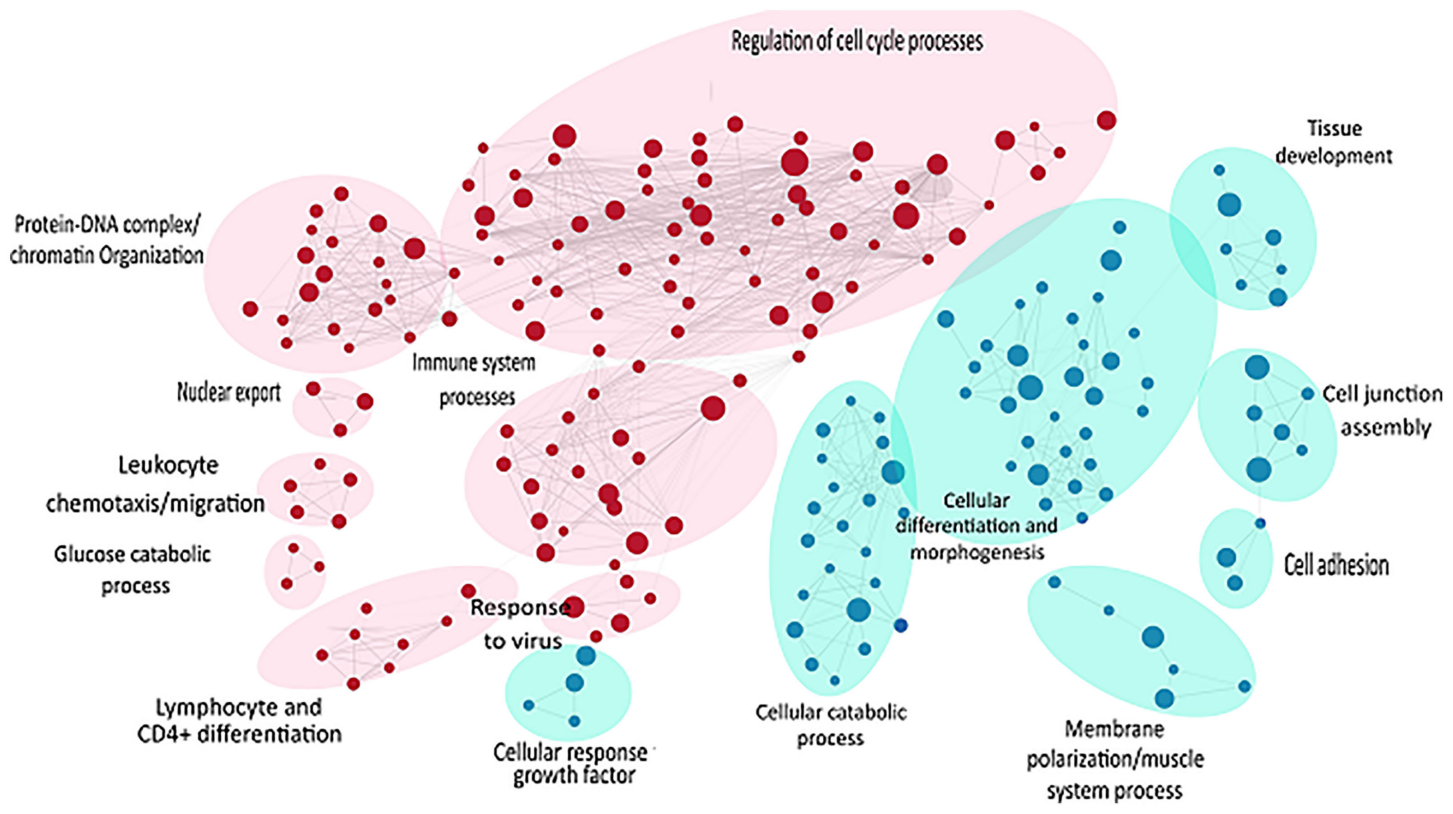
GSEA of raw counts data using Gene Ontology-Biological processes. Pathways upregulated in OS diseased samples are shown in red, while pathways enriched in control samples are shown in blue. Node size indicates size of that gene set, and edge width indicates the similarity coefficient between two gene sets.

Likewise, gene set enrichment analysis using Gene Ontology-Biological processes validated marked upregulation of a diverse range of early immune regulation pathways, leukocyte chemotaxis and migration, lymphocyte and CD4^+^ T cell differentiation, and skin development and differentiation (**Figure 4**). The biological process showed obvious downregulation in cellular catabolic process, tissue morphogenesis, cellular response to growth factors, cellular adhesion, and tissue developmental processes. Densely connected network of GO terms not only indicated the robustness of the analysis but also validated the differential expression-based gene ontology results. GSEA analysis also highlighted multiple metabolic processes along with developmental processes in downregulated pathways (**Figure 4**).

### Pattern of Itch-Associated Genes of Mite-Infested Human Skin Biopsies (GSE178563)

**Figure 5:**
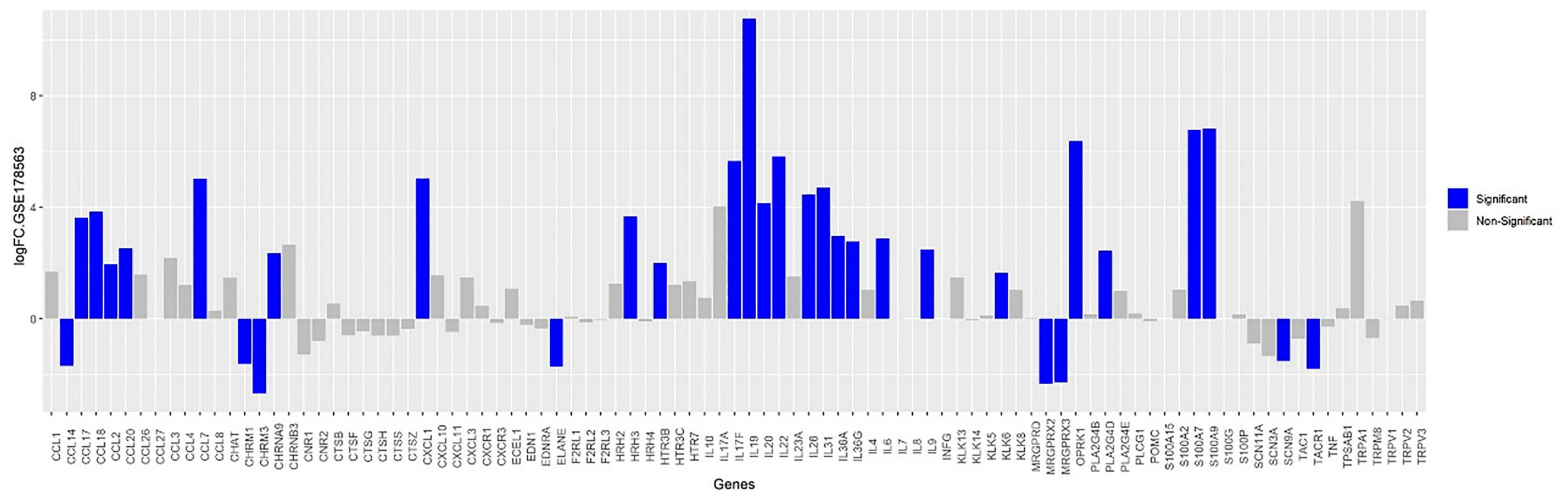
Bar plot of itch-associated genes of GSE178563 (mite-infested human skin biopsies).

DEGs analysis identified itch-associated genes in mite-infested human skin biopsies (GSE178563) as represented in bar plot (**Figure 5**). DEGs in scabies mite-infested skin included those encoding certain chemokines (C-C motif) ligands CCL2, CCL17, and CCL 18, (C-X-C motif) ligand CXCL1, IL-17A, IL-17F, IL-23, IL-31 with FC greater than two for most of the genes. IL-4, CCL7, CCL20, IL-19, IL-20, IL-36A were also prominent DEGs with high FCs that were significantly correlated with the itch severity scores. Phospholipase A2 (PLA2) group IV PLA2G4D and S100A9 and A7 were also increased in itchy skin. In addition, the histamine receptor 3 (HRH3) and serotonin receptor (HTR) 3C were also dominantly upregulated in scabies along with neuropeptide genes that are specifically present in human scabies DEGs list, i.e., Kappa-type opioid receptor OPRK1 and neuronal acetylcholine receptor subunit alpha 9 (CHRNA9) genes involved in neuropeptide signaling pathway and calcium channel activity, respectively.

### Comparative Transcriptomic Analysis

We searched GEO and Array Express databases to identify previously performed gene expression studies on *S. scabiei*–infested samples. Although we did not find any existing transcriptome profiling using human skin biopsies, two *ex-vivo* models on artificially infested porcine samples and human skin equivalents E-MTAB-6433 (23) and GSE48459 (24) were previously compared with the pertinent comparison groups. Herein, we have performed comparative analysis of each of these studies with our model to identify a robust gene signature.

#### I. Association Between Mite-Infested Human Skin Biopsies (GSE178563) and *Ex-Vivo* Porcine Infested Model (E-MTAB-6433)

**Figure 6:**
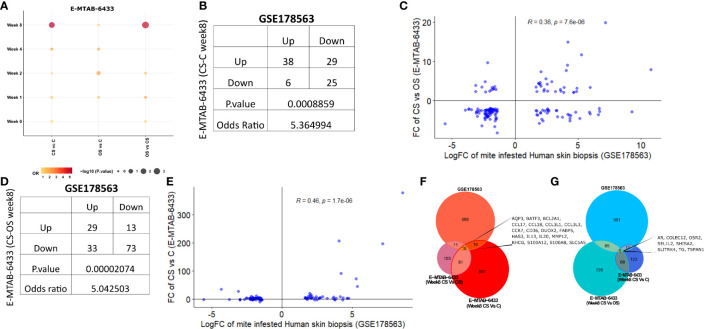
Comparative analysis of GSE178563 (mite-infested human skin biopsies) with E-MTAB-6433 (mite-infested pig biopsies). **(A)** Association between significantly modulated differential expression profiles of human infested skin biopsies with each time point (Week 0,1,2,4,8) of porcine infested groups. Association statistics (contingency table along with odds ratio and fisher’s exact p-value) and correlation plots of significant porcine groups, i.e (1)., CS *vs.* C **(B, C)** and (2) CS *vs.* OS **(D, E)**. Combined Venn diagram of all significantly associated groups identified in **(F, G)** (Week 8 CS *vs.* C and Week 8 CS *vs.* OS mite-infested human samples *vs.* control).

Recently, Bhat et al. employed a porcine model to study time-mediated gene expression profiles from different scabies subtypes. The transcriptomic profile of porcine model was periodically observed over five different time points (Week 0, 1, 2,4, 8) and three different comparison groups, i.e., crusted scabies (CS), ordinary scabies (OS), and control samples (23). The study compared all 15 combinations with the respective controls (i.e., five time points and three comparison groups) and presented the associated list of differentially expressed genes in their research article. Herein, we retrieved the human orthologs of differentially expressed gene lists and performed the association analysis of each comparison from porcine model with mite-infested human biopsies dataset (GSE178563). We identified human orthologs for more than 80% of the porcine differentially expressed genes, 9 to 53% of those retrieved human orthologs also showed significant differential expression in the mite-infested human skin biopsies (**Table 2**). Although earlier time points of porcine model showed no association with human infested samples, two groups of porcine models at week 8 showed a significant positive association with human biopsy dataset, and genes are conserved between human and porcine database (**Figure 6A** and **Supplementary Table 3**).

**Table 2:** Number of differentially expressed genes in mite-infested porcine and human samples.

Porcine comparison groups	Time series	Number of DE genes in mite-infested porcine groups (E-MTAB-6433)	Number of human orthologs (Ensembl Biomart)	% of retrieved human ortholog w.r.t. number of DEGs in porcine group	DEGs in mite-infested human skin biopsies (GSE178563; p<0.05)	% of DEGS in human samples w.r.t. total number of retrieved orthologs
**CS-C**	Week 0	1,552	1,290	83.12%	153	11.86%
	Week 1	1,202	1,015	84.44%	114	11.23%
	Week 2	1,272	1,067	83.88%	105	9.84%
	Week 4	367	306	83.38%	45	14.71%
	Week 8	1,006	842	83.70%	98	11.64%
**OS-C**	Week 0	344	287	83.43%	34	11.85%
	Week 1	952	797	83.72%	75	9.41%
	Week 2	1,955	1,640	83.89%	181	11.04%
	Week 4	758	622	82.06%	84	13.50%
	Week 8	1,573	1,322	84.04%	154	11.65%
**CS-OS**	Week 0	1,297	1,072	82.65%	572	53.36%
	Week 1	1,305	1,074	82.30%	108	10.06%
	Week 2	1,651	1,366	82.74%	148	10.83%
	Week 4	261	217	83.14%	22	10.14%
	Week 8	1,292	1,081	83.67%	149	13.78%

Crusted scabies *vs.* control (E-MTAB-6433) at week 8 exhibited a positive association with human infested biopsies (OR = 5.36; p= 8.86e-04; **Figure 6B**) such that the number of genes dysregulated in the same direction (38 genes upregulated and 25 genes downregulated in both profiles) was significantly more than the genes regulated in the opposite direction (29 genes upregulated in porcine model and downregulated in human skin biopsies; 6 genes downregulated in porcine model and upregulated in human skin biopsies) (**Figure 6B**). Fold change of CS *vs.* OS porcine model also showed a significant positive correlation with mite-infested human dataset (R=0.36; p=7.6e-06; **Figure 6C**). Similar to comparative analysis of CS *vs.* C with mite-infested human biopsies, shared upregulated profile in CS *vs.* OS and human infested biopsies also exhibited marked enrichment of immune response pathways (Cytokine and chemokine signaling, JAK-STAT signaling, IL-4 and IL-13 pathway, and Toll receptor cascade). Likewise, we observed a direct association between CS *vs.* OS (week 8) and mite-infested human skin biopsy samples (OR=5.04; p=2.07e-05; **Figure 6D**). High odd ratio and significant fishers exact test results revealed that the concordant transcriptomic changes (29 shared upregulated and 73 downregulated genes) outnumbered the discordant gene profile (13 genes upregulated in porcine CS *vs.* OS and downregulated in human skin biopsies; 33 genes downregulated in porcine CS *vs.* OS and upregulated in human skin biopsies) (**Figure 6D**). The fold changes also exhibited a linear positive correlation between the two profiles (R=0.46, p=1.7e-06; **Figure 6E**).

Furthermore, we performed the functional analysis using shared gene profiles and found that set of genes upregulated in both profiles [Porcine model CS *vs.* C (Week 8) and human infested skin biopsies] were largely enriched with immune component including “Chemokine signaling pathway, Interleukin-4 and Interleukin-13 signaling, Innate Immune System, Cytokine Signaling in Immune system, and JAK-STAT signaling pathway.” Shared downregulated genes (n = 25) showed an enrichment of PPAR and nuclear signaling pathways. We also observed that genes related to cellular transport, metabolism, and homeostasis were upregulated in porcine CS *vs.* C but downregulated in mite-infested human biopsies (**Table 3**). Contrarily, the downregulated profile did not show enrichment of any signaling pathway, but metabolism of amine-derived hormone was enriched in the discordant gene set (upregulated in porcine and downregulated in humans). Complete data for both porcine model (CS *vs.* C and CS *vs.* OS) merged with the human dataset is available in **Supplementary Tables 4** and **5**, respectively. Unlike the comparison of CS *vs.* OS and CS *vs.* C with human data, the third porcine comparison group (OS *vs.* C) did not show association with human infested biopsies at any given time point, thus indicating a distinct transcriptome profile. Given the shared upregulation of immune component in the three groups, we combined a signature of two significant groups from porcine infested group, i.e., both porcine groups (CS *vs.* OS and CS *vs.* C; Week 8) with human infested samples separately for up- and downregulated genes (**Figures 6F, G**). Herein, we identified that 18 genes were commonly upregulated in all three groups (CS *vs.* C, CS *vs.* OS and human infested skin biopsies; **Figures 6F, G**). As expected, shared upregulated genes primarily included chemokine ligands and receptors (CCL17, CCL18, CCL3L1, CCL3L3, CCR7) and cytokines (IL-13 and IL-20), calcium-binding proteins (S100A2 and S100A8), genes involved in B Cell Receptor Signaling Pathway (sino) and B cell receptor signaling pathway (KEGG) (BCL2A1), fatty acid binding protein in epidermal call and as glycerol transport in skin (FABP5, AQP3, HAS3) and genes that express the cytokines and skin barrier protein in human keratinocytes (S100A12, S100A8) (**Figure 6F**). We observed that eight genes including androgen receptor and negative regulator of Wnt (SHISA2) were commonly downregulated in all three signatures (**Figure 6G**).

**Table 3:** KEGG pathway enrichment of common genes in mite-infested human (Scabies vs. control) and porcine groups (Week 8; CS *vs*. OS and CS *vs*. C).

Comparison Group	Disease condition in Human and Pigs	Gene Set Name	# Genes in Gene Set (K)	Description	# Genes in Overlap (k)	k/K	FDR q-value
**CS Vs. C (Week 8) porcine model and mite infested human skin biopsies**	Commonly Upregulated	GOBP_RESPONSE_TO_ENDOGENOUS_STIMULUS	1624	Any process that results in a change in state or activity of a cell or an organism (in terms of movement, secretion, enzyme production, gene expression, etc.) as a result of a stimulus arising within the organism. [GOC:sm]	9	5.50E-03	1.25E-03
		GOBP_CHEMICAL_HOMEOSTASIS	1187	Any biological process involved in the maintenance of an internal steady state of a chemical. [GOC:isa_complete]	8	6.70E-03	1.25E-03
		KEGG_CYTOKINE_CYTOKINE_RECEPTOR_INTERACTION	265	Cytokine-cytokine receptor interaction	9	3.40E-02	3.79E-09
		REACTOME_SIGNALING_BY_INTERLEUKINS	463	Signaling by Interleukins	9	1.94E-02	2.70E-07
		REACTOME_CYTOKINE_SIGNALING_IN_IMMUNE_SYSTEM	719	Cytokine Signaling in Immune system	10	1.39E-02	4.12E-07
		REACTOME_CHEMOKINE_RECEPTORS_BIND_CHEMOKINES	58	Chemokine receptors bind chemokines	5	8.62E-02	9.73E-07
		KEGG_CHEMOKINE_SIGNALING_PATHWAY	189	Chemokine signaling pathway	6	3.17E-02	7.23E-06
		REACTOME_INTERLEUKIN_4_AND_INTERLEUKIN_13_SIGNALING	111	Interleukin-4 and Interleukin-13 signaling	5	4.50E-02	1.75E-05
		REACTOME_INNATE_IMMUNE_SYSTEM	1117	Innate Immune System	9	8.10E-03	1.48E-04
		REACTOME_PEPTIDE_LIGAND_BINDING_RECEPTORS	198	Peptide ligand-binding receptors	5	2.53E-02	2.33E-04
		REACTOME_NEUTROPHIL_DEGRANULATION	479	Neutrophil degranulation	6	1.25E-02	9.23E-04
		REACTOME_INTERLEUKIN_10_SIGNALING	46	Interleukin-10 signaling	3	6.52E-02	1.88E-03
	Commonly Downregulated	KEGG_PPAR_SIGNALING_PATHWAY	69	PPAR signaling pathway	4	5.80E-02	1.73E-04
		REACTOME_TRANSCRIPTIONAL_REGULATION_OF_WHITE_ADIPOCYTE_DIFFERENTIATION	84	Transcriptional regulation of white adipocyte differentiation	3	3.57E-02	1.74E-02
		REACTOME_SIGNALING_BY_NUCLEAR_RECEPTORS	297	Signaling by Nuclear Receptors	4	1.35E-02	1.93E-02
	Downregulated in Porcine and Upregulated in Humans	No enrichment					
	Upregulated in Porcine and downregulated in Humans	REACTOME_TRANSPORT_TO_THE_GOLGI_AND_SUBSEQUENT_MODIFICATION	186	Transport to the Golgi and subsequent modification	4	2.15E-02	1.70E-02
		REACTOME_THYROXINE_BIOSYNTHESIS	10	Thyroxine biosynthesis	2	2.00E-01	2.01E-02
		REACTOME_TFAP2_AP_2_FAMILY_REGULATES_TRANSCRIPTION_OF_GROWTH_FACTORS_AND_THEIR_RECEPTORS	15	TFAP2 (AP-2) family regulates transcription of growth factors and their receptors	2	1.33E-01	2.69E-02
		REACTOME_ASPARAGINE_N_LINKED_GLYCOSYLATION	305	Asparagine N-linked glycosylation	4	1.31E-02	2.69E-02
		REACTOME_METABOLISM_OF_AMINE_DERIVED_HORMONES	18	Metabolism of amine-derived hormones	2	1.11E-01	2.69E-02
		REACTOME_CLASS_A_1_RHODOPSIN_LIKE_RECEPTORS	331	Class A/1 (Rhodopsin-like receptors)	4	1.21E-02	2.69E-02
		GOBP_ENZYME_LINKED_RECEPTOR_PROTEIN_SIGNALING_PATHWAY	1072	Any series of molecular signals initiated by the binding of an extracellular ligand to a receptor on the surface of the target cell, where the receptor possesses catalytic activity or is closely associated with an enzyme such as a protein kinase, and ending with regulation of a downstream cellular process, e.g. transcription. [GOC:mah, GOC:signaling, ISBN:0815316194]	10	9.30E-03	1.97E-05
		GOBP_TRANSMEMBRANE_RECEPTOR_PROTEIN_TYROSINE_KINASE_SIGNALING_PATHWAY	737	A series of molecular signals initiated by the binding of an extracellular ligand to a receptor on the surface of the target cell where the receptor possesses tyrosine kinase activity, and ending with regulation of a downstream cellular process, e.g. transcription. [GOC:ceb, GOC:signaling]	8	1.09E-02	1.69E-04
		GOBP_REGULATION_OF_CELLULAR_RESPONSE_TO_GROWTH_FACTOR_STIMULUS	296	Any process that modulates the rate, frequency, or extent of a change in state or activity of a cell (in terms of movement, secretion, enzyme production, gene expression, etc.) as a result of a growth factor stimulus. [GOC:tb]	6	2.03E-02	1.79E-04
		GOBP_CELL_MIGRATION	1602	The controlled self-propelled movement of a cell from one site to a destination guided by molecular cues. Cell migration is a central process in the development and maintenance of multicellular organisms. [GOC:cjm, GOC:dph, GOC:ems, GOC:pf, Wikipedia:Cell_migration]	10	6.20E-03	1.79E-04
		GOBP_TRANSMEMBRANE_TRANSPORT	1605	The process in which a solute is transported across a lipid bilayer, from one side of a membrane to the other. [GOC:dph, GOC:jid]	10	6.20E-03	1.79E-04
		GOMF_MOLECULAR_TRANSDUCER_ACTIVITY	1489	A compound molecular function in which an effector function is controlled by one or more regulatory components. [GOC:dos, GOC:pdt]	9	6.00E-03	8.69E-04
		GOBP_LOCOMOTION	1975	Self-propelled movement of a cell or organism from one location to another. [GOC:dgh]	10	5.10E-03	8.69E-04
		GOBP_ION_HOMEOSTASIS	787	Any process involved in the maintenance of an internal steady state of ions within an organism or cell. [GOC:ai]	7	8.90E-03	1.25E-03
**CS Vs. OS (Week 8) porcine model and mite infested human skin biopsies**	Commonly Upregulated	KEGG_CYTOKINE_CYTOKINE_RECEPTOR_INTERACTION	265	Cytokine-cytokine receptor interaction	8	3.02E-02	2.14E-08
		REACTOME_SIGNALING_BY_INTERLEUKINS	463	Signaling by Interleukins	7	1.51E-02	2.83E-05
		REACTOME_CYTOKINE_SIGNALING_IN_IMMUNE_SYSTEM	719	Cytokine Signaling in Immune system	7	9.70E-03	3.69E-04
		KEGG_JAK_STAT_SIGNALING_PATHWAY	155	Jak-STAT signaling pathway	4	2.58E-02	2.07E-03
		REACTOME_CHEMOKINE_RECEPTORS_BIND_CHEMOKINES	58	Chemokine receptors bind chemokines	3	5.17E-02	3.03E-03
		KEGG_CHEMOKINE_SIGNALING_PATHWAY	189	Chemokine signaling pathway	4	2.12E-02	3.03E-03
		REACTOME_THYROXINE_BIOSYNTHESIS	10	Thyroxine biosynthesis	2	2.00E-01	5.73E-03
		REACTOME_TOLL_LIKE_RECEPTOR_TLR1_TLR2_CASCADE	103	Toll Like Receptor TLR1:TLR2 Cascade	3	2.91E-02	1.24E-02
		REACTOME_INTERLEUKIN_4_AND_INTERLEUKIN_13_SIGNALING	111	Interleukin-4 and Interleukin-13 signaling	3	2.70E-02	1.24E-02
		REACTOME_IRAK4_DEFICIENCY_TLR2_4	18	IRAK4 deficiency (TLR2/4)	2	1.11E-01	1.24E-02
	Commonly Downregulated	No enrichment					
	Downregulated in Porcine and Upregulated in humans	No enrichment					
** **	Upregulated in Porcine and downregulated in Humans	REACTOME_METABOLISM_OF_AMINE_DERIVED_HORMONES	18	Metabolism of amine-derived hormones	2	1.11E-01	2.62E-02

#### II. Association of Mite-Infested Human Skin Biopsies (GSE178563) With Human Skin Equivalents (GSE48459)

**Figure 7:**
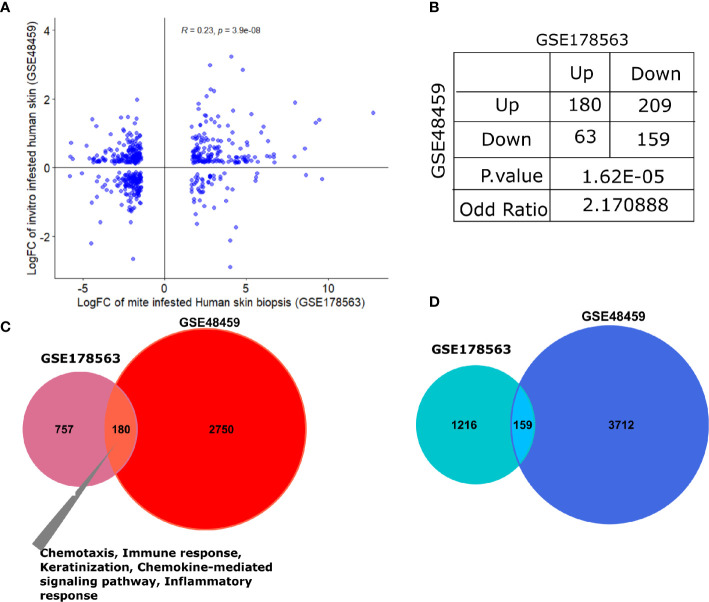
Comparative transcriptomic analysis of GSE178563 (mite-infested human skin biopsies) with GSE48459 (live-mite-infested human skin equivalent group). **(A, B)** Correlation plot of logFC profiles and association statistics (contingency table and fisher’s exact test) of significantly differentially expressed genes in mite-infested human skin biopsies and *in-vitro* human skin equivalents. **(C, D)** Venn diagram of common significantly upregulated and downregulated genes. Significantly enriched pathways in the shared profiles are mentioned.

The human skin equivalents were employed to identify the gene signature that responds to burrowing of scabies mite (GSE48459) (24). Herein, we compared infested human skin equivalents with infested skin biopsy transcriptomics profile (GSE178563). Although the original study compared three different groups—controls, live mites, and mite extract—we employed the live mites *vs.* control group for comparison with our mite-infested human skin biopsy transcriptome (GSE178563). We found a significant positive correlation between logFC of both datasets (R=0.21, p=3e-07; **Figure 7A**). Association analysis between both datasets (skin biopsy and *in-vitro* skin equivalent) aligned with the porcine comparative analysis presented in the previous section. We observed that both human-based transcriptomic profiles exhibited a direct association with an odds ratio of 2.17 (p=1.62e-05; **Figure 7B**), thus indicating that genes dysregulated in the same direction (i.e., concordantly up- or downregulated) were more pronounced than the ones in the opposite direction (**Figure 7B**). As expected, compared to the pig’s dataset, GSE48459 had more genes in common with human skin biopsy dataset (**Figures 7B–D**). One hundred eighty shared upregulated genes were significantly enriched in chemotaxis, chemokine-mediated signaling pathway, inflammatory response, and keratinization (**Figure 7C**). The set of downregulated genes did not show any significantly enriched pathway (**Figure 7D**). The complete merged data of both human (GSE178563) and human skin equivalent (GSE48495) is available in **Supplementary Table 6**.

## Discussion

*S. scabiei* var. *hominis* causes skin infestation in humans by forming burrows in the stratum corneum of the skin and feeding on host epidermis and sera. The release of active metabolites from the mite contribute to the intense itching and inflammation manifesting as a skin allergy and inflammatory responses (2). Potential sources of allergens include fecal material, mite bodies, mite saliva secreted at feeding, soluble proteins released after death in body fluids, and enzymes involved in molting and digestive processes (34). The interactions between host and parasite are of great concern. Our aim was to provide details about disease regulation and development in human scabies by giving an insight into any underlying predisposition for the diseased condition and determine the specific host immune responses and differentially expressed genes after mite infestation. Our study is the first extensive *in vivo* analysis in humans regarding the gene expression profiles in OS. We also compare our data with two scabies-related gene expression datasets, i.e., porcine model (E-MTAB6433) (23) and human skin equivalents (GSE48459) (24).

### Upregulated Immunity Genes

The immunomodulatory effects of *S. scabiei* on the host assists in immune evasion and infestation and can be the possible reason for delayed clinical manifestation during primary infestation (35). The top upregulated gene associated to have a role in inflammatory responses in OS is DEFB4A, which is known to be induced by IL-17A in epidermal keratinocytes and reconstituted human epidermis (36, 37). Increased expression of DEFB4A (IL-17 signature gene) is linked with an increased number of CD3+, CD8+ T cells in psoriasis (38, 39) and reported to play an important role in the onset of asthma and atopy (40).

IL-19 is reported to have role in inflammation, vascularization, and tissue remodeling (41), and associated with induced Th2 cytokines in allergic patients. IL-19 binds the dimer receptor IL-20R leading to activation of the JAK/STAT signaling pathway (42). Additionally, it has been suggested IL-19 might act as an assessment tool for psoriasis and atopic dermatitis patients (43), and along with IL-23/IL-17, has strengthened its role as a biomarker for chronic inflammatory disorders (44).

CXCL8-encoded IL-8 was also upregulated in OS and has roles in inflammatory cell activation, initiation of inflammatory responses, and migration of neutrophils to sites of inflammation (45, 46). CXCL8 receptor expression has been mainly found in psoriasis keratotic lesions and contributes to severity of psoriasis by the release of inflammatory mediators and migration of neutrophils to lesion site (47, 48). Neutrophils initiate inflammation and mediate tissue destructive events in several inflammatory diseases (49, 50). Neutrophils were reported as prominent inflammatory cell infiltrate in cases of human scabies as well as scabies-infested wombats, sheep, and red foxes (51, 52).

CSF3 belongs to the IL-6 superfamily and is among the upregulated genes of OS. CSF3 has been shown to have a role in the inflammatory response, cytokine-mediated signaling pathways, and signal transduction, and known to be a factor involved in immune cell activation and recruitment in *Psoroptes ovis* (*P. ovis*) (21). CSF3 was one of the upregulated genes differentially expressed by human skin equivalents exposed to live scabies mites (24).

The SERPINB4 gene is upregulated in OS, which suggests it is more likely to be the part of acute response rather than a chronic effect (53–55). SERPINB4, first identified for its protease inhibition activity, has also been reported to have roles in controlling important innate immune pathways involving clotting, inflammation, and complement cascade (56). Interestingly, SERPINB4 is also found secreted in *Sarcoptes* mite gut and excreted out with mite feces into formed burrows. It has been shown to promote the growth of group A *streptococcus* (GAS) and *S. aureus*. This colonization of mite-infected skin with GAS, *S. aureus*, and other pathogens could be the reason of systemic infection and may lead to deleterious outcomes in patients with severe scabies (57). Additionally, other studies also reported the increased expression of SERPINB4 gene in skin and serum of psoriasis patients (58, 59).

Also included among the top upregulated DEGs is S100A7A. This has previously been identified among psoriasis-associated genes involved in activation and proliferation of keratinocytes, modulation of host immune response, and antimicrobial defense (59), and as a biomarker in atopic dermatitis (60), and also upregulated in scabies inflammatory processes (Bhat et al., 2020). S100A7A (psoriasin) is an antimicrobial peptide that also has roles in migration of keratinocytes, dendritic cells, and T lymphocytes and the activation of the innate and acquired immune response (61, 62).

HRNR, another upregulated gene of OS, has been recently reported to be considered a biomarker for differential diagnosis of atopic dermatitis (63) and expressed in human epidermal keratinocytes (64). GWAS (genome-wide association study) also revealed that upregulation of HRNR gene is one of the stronger risk factors for eczema, more than for hay fever or asthma (65). HRNR contributes to antimicrobial and protective functions in healthy skin, and its reduced expression is reported to be associated with epidermal barrier defects in atopic dermatitis infection (66).

Differentially expressed genes that were enhanced in OS included immune-related signaling pathways and JAK-STAT pathways. Their impacts include activation of macrophages and neutrophils, host inflammatory responses, and the differentiation of B and T cells, which control the wound repair (67). The JAK-STAT signaling pathway has been noted in rabbits infested with *S. scabiei*, where it was differentially expressed at multiple time points, indicating the main signaling pathway of innate immune response (23, 68). JAK1 and JAK2 signaling pathways are involved in dysregulation in atopic dermatitis and asthma immune response and include Th2 response exaggeration, B-cell maturation, and activation of eosinophils. It is crucial in atopic dermatitis pathogenesis with upregulation of epidermal chemokines and pro-inflammatory cytokines and downregulation of antimicrobial peptides. Thus, it plays a key role in the pathogenesis of immune-mediated disease and design of novel therapeutic approaches in the treatment of immune disorders (69, 70).

### Upregulated Genes for Keratinization

Keratinocyte dermal cells has been reported to modulate the secretion of cytokines and expression of cell adhesion molecules in response to scabies mite infestation (24). The top six upregulated genes for keratinization in OS include KRT6C, which is also reported to be highly upregulated gene in psoriatic skin (59, 71, 72), and KRT9 gene, a mutation that is responsible for the most common form of autosomal dominant Palmoplantar keratodermas (PPK) (73, 74). LCE3A gene is also associated with keratinization and was upregulated in OS. It is associated with cornification of the epidermis to become stratum corneum in human skin equivalents when exposed with scabies live mites (24), have defensin-like antimicrobial activity against a variety of bacterial taxa (75, 76), have role in skin repair, and reported to be upregulated in psoriasis (77, 78). The upregulated OS genes SPRR3 and SPRR2A are the encrypt for the Late Cornified Envelope (LCE) protein present in cornified cell envelope (CE) in both psoriasis and atopic dermatitis; the expression of these genes is linked to keratinocyte terminal differentiation both *in vivo* and *in vitro* (79).

Wnt signaling pathway is involved at the earliest stage of skin development as a dominant pathway controlling the patterning of the skin, cell proliferation, and maintaining homeostasis of the skin (80–82). The downregulation of Wnt signaling pathways has been observed in OS samples and corresponds to pathway analysis in psoriasis, which revealed downregulation of all members of the canonical Wnt signaling pathway (83).

### Top Downregulated Genes

Our results revealed that amongst the top downregulated genes, the antigen recognition and binding of immunoglobulin genes (IGLV6-57, IGHV3-30, IGLV4-69, IGHEP1) and T-cell receptor gene (TRBV13) in OS samples are similar to that reported previously (23). These genes were shown to be downregulated in the porcine model of CS at 2 and 8 weeks, where antigen-presenting cells are essential for T cell activation. Conversely, upregulation of immunoregulatory molecules in mite infestation may suppress the pathogenic inflammatory T cells, which contribute to skin pathology in OS.

AADACL3 gene, reported to be amplified in recurrent crusted scabies samples, was found among the most deregulated genes in our study. AADACL3 has duplicate regions on chromosome 1 (1p36) and belongs to a lipolytic enzyme family. Its significance in skin immunodeficiency is not still clear, but it has been reported that the duplicated regions contained more than 100 genes downregulated at the mRNA level in unaffected tissue (33).

Our results showed that the pathways associated with skin development, including keratinization, epidermis development, and keratinocyte differentiation, were highly upregulated in OS, which is also in accordance with previous findings (24). In addition, early immune-related responses along with cytokine activity, chemokine signaling pathways, monocyte chemotaxis, and CXCR (chemokine receptor binding) are also upregulated in scabies transcriptomic profiles. The immune component has previously been shown to be involved in scabies infestation (84), and particularly cytokines along with chemokines orchestrate this early immune response in scabies (35).

### Itch-Associated Genes of Mites

The PLA2 family of group IV enzymes are involved in cell signaling and the inflammatory response *via* production of arachidonic acid, which is a precursor for eicosanoids. The eicosanoid subfamily of prostaglandins and leukotrienes is known to be involved in itch (85). Furthermore, TRPV1, which mediates histamine-induced itching *via* activation of PLA2, was significantly downregulated in scabies itch (86, 87). The k-opioid receptor gene (OPRK1) was upregulated, which may play a significant role in the propagation of chronic itch (88). Another promising target is HRH3, the gene for histamine receptor 3. This was found to be overexpressed in scabies mite-infested skin. It is involved in enhancement of antigen-presenting capacity of dendritic cells and TH1priming. H3Rs are also reported to have a role in treatment of some allergic and inflammatory conditions. H3R is also responsible for the allergic rhinitis symptoms, atopic dermatitis, and pruritus (89, 90). IL-4 and IL-31 were also significantly high in scabies mite-infested skin. IL-4 is reported to increase the itching even in low dose of histamine (91), and IL-31 is known to be involved in causing itching by signal activation of IL-13 receptors, but the mechanism involved is still unclear (92). Moreover, JAK1 signaling in sensory neurons also leads to chronic itching, and blocking of neurol JAK1 signaling can limit itching in non-inflammatory situation (91).

### Comparative Analysis of Mite-Infested Human Skin Biopsies

Comparative analysis between mite-infested human skin biopsies and E-MTAB6433 (crusted scabies *vs.* control; week 8) revealed a direct association between porcine crusted scabies *vs.* ordinary scabies (week 8) as well as for both pig groups (CS *vs.* OS and CS *vs.* C; Week 8). The shared upregulated genes between mite-infested human skin biopsies and porcine database include chemokine ligands and receptors (CCL17, CCL18, CCL3L1, CCL3L3, CCR7), which play a crucial role in immune cell activation and stimulation of host inflammatory responses. CCL17 and CCL18 are chemokines that are significantly involved in T cell–mediated reactions and characteristic for various inflammatory skin diseases with TH2 dominance (93). CCL3L1 and CCL3L3 caused inflammatory cellular infiltrate in burrowing mites, and effector molecules are associated with salivary secretion and fecal material and reported to induce chemotactic response in the mite vicinity (24). Likewise, several chemokines, CCL3L1, CCL17, CXCL2, and selectin SELPLG, were upregulated at week 8 in CS and have been implicated in various inflammatory skin diseases. Whereas CXCL11 and CXCL16 are associated with chemotactic T-cell activation in skin and were downregulated at week 8 in CS (23).

The human skin equivalents study also showed upregulation of cutaneous T cell-attracting chemokine (CTACK, CCL27) and thymus- and activation-regulated cytokine (TARC, CCL17) in response to scabies mites (94). The reported list of inflammatory mediators found in atopic dermatitis includes S100A7, S100A8 S100A9, CCL2, CCL3, IL36A, IL36G, and IL36RN. These are involved in receptors triggering expression of myeloid cells TERM1 and skin barrier proteins and keratin 16 (95).

The cytokines IL-13 and IL-20 were also upregulated in all three groups studied in comparison analysis. IL-13 is known to drive antibody class switching and induce expression of IgG_4_ (96). It has been associated with Th2 type inflammation in scabies inflammatory and allergic responses (97), have a role in macrophage activation (98), and contribute to allergic inflammation in CS (23, 35). IL-20 is also a proinflammatory cytokine produced by keratinocytes, monocytes, and endothelial cells and is associated with epidermal thickening, scales, and crusts in CS (19, 99). The analysis of KEGG pathway at week 8 showed upregulation of JAK-STAT signaling pathway, as reported previously, and in the CS *vs.* C and CS *vs.* OS commonly upregulated groups (46).

Our transcriptomic data analysis of mite-infested human skin biopsies in OS *vs.* C revealed genes with pathophysiology of inflammatory disorders as seen in psoriasis and atopic dermatitis. The result has provided a significant insight into inflammatory and susceptible immune disorders responsible in scabies and the role of these chemokines, cytokines, and certain other molecules and related pathways in scabies clinical manifestations. Our study has provided an insight into cytokine-mediated signaling pathways and signal transduction. These include genes involved in immune cell activation and recruitment in OS, including DEFB4A and IL-19 induced by IL-17A, which cause upregulation of Th2 cytokines in allergic patients. IL-19 may also be applicable as an assessment tool in scabies, as suggested for psoriasis and atopic dermatitis patients. SERPINB4 genes play a role in inflammatory responses and control the important innate immune responses involving inflammation and complement cascade. The differentially expressed genes of OS are immune-related signaling pathways enriched in JAK-STAT pathways, which impact activation of macrophages and neutrophils, host inflammatory responses, and regulate the differentiation of B and T cells. IL-17 is a promising immunotherapeutic target and could be a promising therapy for the treatment of OS, in combination with acaricides. Immune-based therapies are currently in clinical trials for various inflammatory diseases, and efficacy has been seen in clinical trials in psoriasis.

The detailed transcriptomic profile has provided an insight into molecular functions, biological processes, and immunological responses. It has increased our understanding about overall gene expression in scabies in human, identified the crucial underlying biological process involved in scabies pathology and physiology, and identified immunotherapy targets for scabies.

## Data Availability Statement

The data set of this research work have been deposited in NCBI’s Gene Expression Omnibus and are accessible through GEO Series accession number GSE178563 (https://www.ncbi.nlm.nih.gov/geo/query/acc.cgi?acc=GSE178563).

## Ethics Statement

Ethical approval for the collection of human skin biopsies was granted by the Chairman, Institutional Review Board, and Ethics Committee of the National University of Medical Sciences (NUMS), Rawalpindi, Pakistan, after compliance with the observations of the two reviewers, under letter No. NUMS/P(VC) – 17/R&D/ORIC/IRB&EC approved on November 9, 2017. The patients/participants provided their written informed consent to participate in this study.

## Author Contributions

SN and SW conceived, designed, and wrote and reviewed the project and manuscript. HS, SI, and AF conducted the analysis and also wrote part of the manuscript. WH provided the technical facilities and helped in analysis. ZS provided the samples and data for the study. All authors contributed to the article and approved the submitted version.

## Funding

The project is funded by Higher Education Commission (HEC), Pakistan, under Startup Research Grant Program (SRGP) to SN (SRGP # 1640). The funders had no role in study design, data collection and analysis, decision to publish or preparation of the manuscript.

## Conflict of Interest

The authors declare that the research was conducted in the absence of any commercial or financial relationships that could be construed as a potential conflict of interest.

## Publisher’s Note

All claims expressed in this article are solely those of the authors and do not necessarily represent those of their affiliated organizations, or those of the publisher, the editors and the reviewers. Any product that may be evaluated in this article, or claim that may be made by its manufacturer, is not guaranteed or endorsed by the publisher.

## Supplementary Material

Supplementary Table 1 Clinical and demographic profile of human skin biopsy samples in GSE178563.

Supplementary Table 2 Number of RNA-seq reads per sample.

Supplementary Table 3 Association analysis (contingency tables, odds ratio, and fisher’s exact test) of mite-infested human scabies with 15 different porcine groups.

Supplementary Table 4 DEGs in porcine group (CS *vs.* C; Week 8) and matched differential expressed results from mite-infested human dataset.

Supplementary Table 5 DEGs in porcine group (CS *vs.* OS; Week 8) and matched differential expressed results from mite-infested human dataset.

Supplementary Table 6 Complete differential expression profile from mite-infested human skin equivalents (GSE48456) and human skin biopsies (GSE178563).

Supplementary Figure 1 RNA integrity (RIN) values of eight samples used for RNA sequencing.
