# Supplementary figures and images for: Transcriptome Analysis of Host Inflammatory Responses to the Ectoparasitic Mite Sarcoptes scabiei var. hominis
Source: Front Immunol. 2021 Dec 1;12:778840. doi: 10.3389/fimmu.2021.778840 (PMC8671885; doi:10.3389/fimmu.2021.778840)

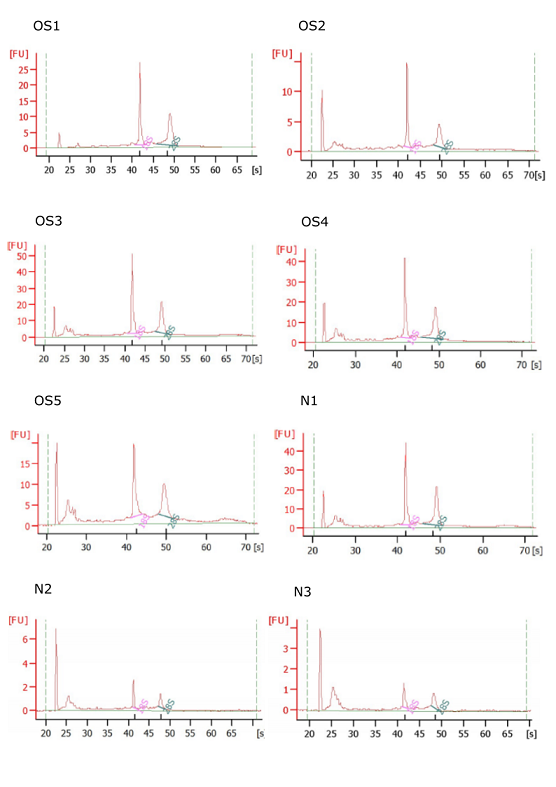

Supplement: Supplementary Figure 1 — RNA integrity (RIN) values of eight samples used for RNA sequencing. [file Image_1.tif]
